# Alpha-hederin reprograms multi-miRNAs activity and overcome small extracellular vesicles-mediated paclitaxel resistance in NSCLC

**DOI:** 10.3389/fphar.2024.1257941

**Published:** 2024-02-01

**Authors:** Yuzhen Chang, Xinyu Gao, Yuchen Jiang, Jingyi Wang, Liu Liu, Jun Yan, Gang Huang, Hao Yang

**Affiliations:** ^1^ Shanghai Key Laboratory of Molecular Imaging, Jiading District Central Hospital Affiliated Shanghai University of Medicine and Health Sciences, Shanghai, China; ^2^ Graduate School, Shanghai University of Traditional Chinese Medicine, Shanghai, China; ^3^ School of Health Science and Engineering, University of Shanghai for Science and Technology, Shanghai, China; ^4^ Department of Nuclear Medicine, Shanghai Chest Hospital, School of Medicine, Shanghai Jiao Tong University, Shanghai, China; ^5^ Department of Oncology, Jiading District Central Hospital Affiliated Shanghai University of Medicine and Health Sciences, Shanghai, China

**Keywords:** alpha-hederin, multi-miRNAs, small extracellular vesicles, paclitaxel resistance, NSCLC

## Abstract

**Background:** Small extracellular vesicles (sEVs) mediate intercellular communication in the tumor microenvironment (TME) and contribute to the malignant transformation of tumors, including unrestricted growth, metastasis, or therapeutic resistance. However, there is a lack of agents targeting sEVs to overcome or reverse tumor chemotherapy resistance through sEVs-mediated TME reprogramming.

**Methods:** The paclitaxel (PTX)-resistant A549T cell line was used to explore the inhibitory effect of alpha-hederin on impeding the transmission of chemoresistance in non-small cell lung cancer (NSCLC) through the small extracellular vesicles (sEVs) pathway. This investigation utilized the CCK-8 assay and flow cytometry. Transcriptomics, Western blot, oil red O staining, and targeted metabolomics were utilized to evaluate the impact of alpha-hederin on the expression of signaling pathways associated with chemoresistance transmission in NSCLC cells before and after treatment. *In vivo* molecular imaging and immunohistochemistry were conducted to assess how alpha-hederin influences the transmission of chemoresistance through the sEVs pathway. RT-PCR was employed to examine the expression of miRNA and lncRNA in response to alpha-hederin treatment.

**Results:** The resistance to PTX chemotherapy in A549T cells was overcome by alpha-hederin through its dependence on sEV secretion. However, the effectiveness of alpha-hederin was compromised when vesicle secretion was blocked by the GW4869 inhibitor. Transcriptomic analysis for 463 upregulated genes in recipient cells exposed to A549T-derived sEVs revealed that these sEVs enhanced TGFβ signaling and unsaturated fatty acid synthesis pathways. Alpha-hederin inhibited 15 types of unsaturated fatty acid synthesis by reducing the signaling activity of the sEVs-mediated TGFβ/SMAD2 pathway. Further, we observed that alpha-hederin promoted the production of three microRNAs (miRNAs, including miR-21-5p, miR-23a-3p, and miR-125b-5p) and the sorting to sEVs in A549T cells. These miRNAs targeted the TGFβ/SMADs signaling activity in sEVs-recipient cells and sensitized them to the PTX therapy.

**Conclusion:** Our finding demonstrated that alpha-hederin could sensitize PTX-resistant NSCLC cells by sEV-mediated multiple miRNAs accumulation, and inhibiting TGFβ/SMAD2 pathways in recipient cells.

## 1 Introduction

Lung cancer is the world’s highest incidence and mortality of malignant tumors, among which non-small cell lung cancer (NSCLC) accounts for about 80%. However, chemotherapy resistance is an important cause of treatment failure and recurrence in tumor patients, which hinders effective treatment and patient prognosis (Zhang et al., 2023b). Therefore, the exploration of reversing the resistance of chemotherapy and the mechanism of drug resistance has become a key issue in cancer research.

Small extracellular vesicles (sEVs), also known as exosomes, are tiny extracellular vesicles secreted by cells ranging from 30 to 200 nm in diameter. sEVs are secreted by cancer cells and a variety of stromal cells in the tumor microenvironment, regulate the development and treatment sensitivity of tumors by participating in the transmission of information between cells and controlling signaling pathways (Filipazzi et al., 2012). sEVs increase chemotherapeutic drug resistance by transferring their cargos, including DNA, RNA, proteins and lipids, to cancer and non-cancer cells (Mashouri et al., 2019). The resistance to tumor chemotherapy mediated by sEVs encompasses multiple mechanisms. sEVs, which encapsulate the drug efflux transporter MDR1, played a role in augmenting the resistance of breast cancer to doxorubicin, through an increase in the extracellular efflux of the drug (Wang et al., 2016). Survivin and other anti-apoptotic proteins were notably concentrated within sEVs originating from pancreatic adenocarcinoma. Their presence significantly contributed to the resistance observed against the therapeutic impacts of paclitaxel and gemcitabine (Aspe et al., 2014; Chang et al., 2021). The elements within the tumor microenvironment, notably tumor-associated fibroblasts, released sEVs that harbor a diverse range of microRNAs (miRNAs). This secretion process actively contributed to the development of resistance in colorectal cancer, gastric cancer, pancreatic cancer, and other malignancies against chemotherapy agents like 5-fluorouracil (5-FU), cisplatin, oxaliplatin, and gemcitabine (Hu et al., 2019a; Liang et al., 2020; Zhang et al., 2020; Qi et al., 2023). Our previous studies found that sEVs secreted by resistant cells carries metabolic enzymes to sensitive tumor cells and increases resistance in sensitive cells (Wang et al., 2021a; Yang et al., 2021a; Dai et al., 2022). Thus, as a potential strategy to reverse drug resistance, targeting sEVs pathways may interfere with tumor metabolism and restore sensitivity to chemotherapy agents.

In recent years, traditional Chinese medicine (TCM) monomer components have been used to assist chemotherapy drugs to produce antitumor effects (Feng et al., 2020). However, the mechanism of these components needs to be further explored by means of tumor pharmacology and molecular biology. α-Hederin, a monosaccharide triterpenoid saponins, with chemical formula C_41_H_66_O_12_, are found in Pulsatile anemone and have antitumor effects. Several studies including our group have demonstrated the activity of α-hederin against tumor proliferation and metastasis in multiple types of tumor cells (Cheng et al., 2014; Deng et al., 2019; Fang et al., 2021; Wu et al., 2022b; Chen et al., 2022). To overcome chemotherapy resistance, α-hederin was utilized in both *in vitro* and *in vivo* settings to reverse resistance in NSCLC, gastric cancer and colorectal cancer against cisplatin, paclitaxel and doxorubicin. This reversal was achieved by promoting the accumulation of reactive oxygen species (ROS) and facilitating the breakdown of mitochondrial membrane potential (Kuete et al., 2014; Zhan et al., 2018; Deng et al., 2019). We aim to investigate the potential of α-hederin in overcoming chemotherapy resistance in NSCLC, with a specific focus on addressing sEVs-mediated resistance.

Paclitaxel (PTX, or Taxol) is one of the main chemotherapy drugs in NSCLC, which inhibits the stability of microtubules and induces cell mitotic arrest to eliminate rapidly proliferating tumor cells (Lu et al., 2022; Wang et al., 2022). However, the development of PTX resistance and adverse reactions limit the clinical use. Here, we found that α-hederin increased sensitivity to PTX chemotherapy by blocking sEVs pathways. Mechanistically, α-hederin affected lipid metabolism and TGFβ pathways by regulating vesicular miRNAs, thereby blocking the transmission of sEVs-dependent resistance.

## 2 Materials and methods

### 2.1 Cell lines and reagents

The A549 cell line was purchased from the American Type Culture Collection (ATCC) and cultured in high-glucose DMEM (11965092, GIBCO, NY, USA) containing 10% fetal bovine serum (40131ES76, Yeason, Shanghai, China). PTX-resistant A549 cell lines (A549T) were obtained after progressive exposure to increasing concentrations of PTX for 6 months. The following reagents were purchased: PTX for cell experiments (S1150, Selleck Chemicals, Shanghai, China), PTX for animal experiments (H20171227, TAXOL, Bristol-Myers Squibb, NY, USA), α-hederin (HY-N0255, MedChemExpress, NZ, USA), ITD-1 (S6713, Selleck Chemicals), and GW4869 (S7609, Selleck Chemicals).

### 2.2 Vesicles isolation and identification

The isolation and identification analysis of sEVs was performed following the recommended procedures outlined in MISEV2018 guidelines (Alzahrani et al., 2023). A549T and A549 cells were cultured in serum-free DMEM medium for 72 h. The medium was collected, centrifuged 2000 *g* and 10000 g for 10 min (min) each to remove cellular debris, followed by filtration through a 0.45 μm filter. The culture medium was centrifuged (Optima XPN-100, Beckman Coulter, CA, USA) at 120,000 g for 90 min (4°C) to obtain the precipitate of sEVs. The sEVs precipitate was resuspended in 20 mL PBS, followed by another centrifugation at 120,000 g (4°C) for 90 min, and this process was repeated once. The final obtained sEVs precipitate was suspended in PBS and stored at −80°C. The concentration, size distribution and zeta potential of sEVs were identified using nanoparticle tracking analysis (ZetaView PMX 110, Particle Metrix, Meerbusch, Germany) and ZetaView 8.04.02 software. Specifically, NanoStandards standard particles (700074, Particle Metrix) were injected into the machine for size calibration, followed by the analysis of diluted exosome samples. The NTA settings were configured with parameters as follows: sensitivity = 75, min bright = 20, min area = 5. The morphology of sEVs was identified by transmission electron microscopy (TEM). Briefly, the sEVs solution was placed on a copper mesh plate (BZ110125, Zhongjingkeyi Technology EMCM, Beijing, China) and negatively stained with uranium acetate. The sEVs were then observed using a TEM device (FEI Tecnai G2 Spirit TEM, USA).

### 2.3 Cell viability assay

The supernatant was obtained from 5 × 10^5^ A549T or A549 cells cultured in 1 mL serum-free DMEM medium for 48 h (h). A549 cells were co-cultured with the supernatant medium at 10^4^ cells/mL for CCK-8 assays (40203ES80, Yeason). The absorption value at 450 nm was used to measure cell viability. For sEVs treatment, A549 cells were co-cultured with 10^8^ or 10^9^ particles/mL sEVs or 20 ng/mL PTX, and cell viability was calculated by CCK-8 assays. In order to quantitatively evaluate the effects of combined PTX and α-hederin treatment, A549 and A549T cells were subjected to 0.1–100 ng/mL PTX and 5–20 μg/mL α-hederin, respectively. The inhibition rates resulting from individual treatments and the combined of both drugs were entered into the CompuSyn software (version 1.0.1), utilizing the classic Chou-Talalay method for drug synergy analysis according to the designer’s instructions (Chou, 2010). A combination index value below 1 was regarded as indicative of a synergistic sensitizing effect.

### 2.4 Cell apoptosis assay

A549 or A549T cells at 3×10^5^ in 12-well plates were treated with sEVs, PTX, or α-hederin for 48 h. Cell apoptosis was detected using propidium iodide (PI)-Annexin V apoptosis detection kit (556547, BD Biosciences, NZ, USA) and flow cytometry (Novocyte, Agilent Technologies, CA, USA). NovoExpress software (version 1.5.0, Agilent Technologies) was used to analyze the proportion of apoptotic cells.

### 2.5 RNA sequencing

After treatment of A549 cells with 10^9^ particles/mL of A549T-derived sEVs for 48 h, total RNA was extracted through the tissue/cell RNA miniprep kit (R6311, Biomiga, Hangzhou, China). A total of 500 ng RNA was used for RNA sequencing through the Illumina HiSeq X platform. The sequencing data were obtained as high-quality clean reads using fastp software (v0.20.0). Clean reads were compared with the human reference genome (GRCh38/hg38) using STAR (v2.7.9a), and read counts were obtained as mRNA expression levels using HTSeq software (v0.13.5). Differential mRNA analysis and its Gene Ontology (GO) and Kyoto Encyclopedia of Genes and Genomes (KEGG) pathways analysis were conducted on the Omicsmart online platform (omicsmart.com; Guangzhou Gene *denovo* Biotechnology Co., Ltd., Guangzhou, China). All the detection procedures were performed by Guangzhou Gene *denovo* Biotechnology Co., Ltd. The raw data is available in the SRA database (BioProject ID PRJNA946809).

### 2.6 Western blot

For cells subjected to drug or sEV treatment, 8 × 10^5^ cells cultured in a 6-well plate were lysed using RIPA buffer to extract proteins. Each experiment was replicated three times, and samples were collected accordingly. Proteins in the lysates were electrophoretically separated by SDS-PAGE. The proteins were transferred to PVDF membranes (IPVH00010, Millipore, MA, USA), which were then blocked in a buffer solution containing 5% milk for 1 h. The membrane was incubated overnight at 4 °C with primary antibodies, including SREBF1 (A15586, Abclonal, Wuhan, China), FASN (A0461, Abclonal), phospho-SMAD2 S245/S250/S255 (AP1338, Abclonal), SMAD2 (A19114, Abclonal), TGFβ1 (A2124, Abclonal), CD63(A19023, Abclonal), GRP94 (60012-2-Ig, Proteintech, Wuhan, China), TSG101 (72312, Cell Signaling Technology, MA, USA), and β-tubulin (2128, Cell Signaling Technology). The membranes were washed with TBST to remove non-specific bindings, then incubated with HRP-labeled secondary antibody (abs20147 and abs20163, Absin, Shanghai, China) for 1 h. The electrochemiluminescence method was used to display signals on the membrane, and images were obtained using a chemiluminescence imager (Tanon 5200 multi, Shanghai, China). The ImageJ software (version 1.53, National Institutes of Health, USA) was utilized for the analysis of band density in Western blot images. The measured Integrated Density values were used to quantify the image density for each protein. Subsequently, normalization was performed using an internal reference protein to calculate the relative expression levels of other proteins.

### 2.7 Oil red O stain

A549 or A549T cells cultured in 12-well plates were washed with PBS and stained with oil red O according to the manufacturer’s instructions (D027, Jiancheng Bioengineering, Nanjing, China). The plates were washed with PBS and dried, and the stained cells were observed and photographed under a microscope.

### 2.8 LC/MS assays of free fatty acids

A total of 10^7^ cells were re-suspended in an aqueous solution containing 60% methanol pre-cooled at −40°C. Cell precipitates were collected by centrifugation and rapidly frozen in liquid nitrogen. Each sample was lyzed and centrifuged in 500 μL cold methanol and 1 mL chloroform. The chloroform solution was dried in a rotary evaporator and redissolved in 100 μL methanol. The solution was analyzed using liquid chromatography/mass spectrometry (LC/MS) for its free fatty acids content. The LC was performed using an Acquity UPLC system (Waters, MA, USA), and the MS using AB SCIEX 5500 QQQ-MS (AB SCIEX, ON, Canada). The chromatographic columns used were Acquity UPLC BEH C18 (Waters). The column temperature was 40 °C, and the flow rate was 0.30 mL/min in ultra-performance liquid chromatic-triple quadrupole tandem mass spectrometry (UPLC-QQQ-MS). The mobile phase consisted of water/acetonitrile (35:65, 5 mM ammonium formate) and isopropyl alcohol/acetonitrile (65:35, 5 mM ammonium formate). The content of each fatty acid was calculated using MultiQuant software (version 3.0.2, AB SCIEX). This detection procedure was performed by Guangzhou Gene *denovo* Biotechnology Co., Ltd. (Guangzhou, China).

### 2.9 Development and imaging of xenograft tumors in mice

A total of 5 × 10^6^ A549T cells expressing luciferase were implanted subcutaneously in 30 nude mice, and tumor volume was recorded every 3 days. When the tumor size reached 100 mm^3^ around the 10th day, the solvent control (0.1% DMSO), 3 μg/kg PTX, 5 mg/kg α-hederin, 3 μg/kg PTX +5 mg/kg α-hederin, 3 μg/kg PTX +1 mg/kg GW4869 or 3 μg/kg PTX +5 mg/kg α-hederin +1 mg/kg GW4869 were administered every 3 days by intraperitoneal injection, due to its high bioavailability and close proximity to the subcutaneous tumor location. For bioluminescence imaging, nude mice were intraperitoneally injected with luciferin sodium salt solution (40901ES01, Yeason) at a concentration of 10 mL/kg, and the images were developed using a bioluminescence imager (IVIS SPECTRUM, Perkin-Elmer, MA, USA). For microPET/CT, 200 μCi ^18^F-FDG imaging agent was injected through the tail vein, and nuclear medicine imaging by nanoScan PET/CT (Mediso, Budapest, Hungary) was performed after 45 min. All experimental procedures were approved by the Animal Ethics Committee of Shanghai University of Medicine and Health Sciences.

### 2.10 Immunohistochemistry

The A549T xenograft tumors were fixed in a 4% paraformaldehyde solution followed by paraffin embedding. The tissue morphology was observed by hematoxylin-eosin (HE) staining. For immunohistochemistry (IHC), paraffin sections were treated with 3% H_2_O_2_, blocked with BSA, and incubated overnight with primary antibodies, including TSG101, SREBF1, FASN, p-SMAD2, SMAD2, and TGFβ1. The sections were then incubated with the enzyme-labeled secondary antibody for 1 h, and the positive signal was displayed with diaminobenzidine (DAB). The evaluation of all IHC staining was carried out by two independent researchers to quantitatively analyze each protein. The percentage score for stained cells was categorized as follows: 0 (0%–5%), 1 (5%–25%), 2 (25%–50%), and 3 (50%–100%). Simultaneously, staining intensity was graded on a scale of 0 (no staining), 1 (weak), 2 (moderate), and 3 (strong). The IHC score for each sample was calculated by multiplying the percentage score with the staining intensity score.

### 2.11 RT-qPCR and miRNA transfection

Total RNA from drug-treated A549 or A549T cells was obtained using a cell/tissue RNA isolation kit. Total RNA from A549 or A549T cell-derived sEVs was extracted using a miRNeasy Mini Kit (217084, Qiagen, Hilden, Germany). The first strand cDNA synthesis for lncRNA (R312, Vazyme, Nanjing, China) and miRNA (MR101, Vazyme) detection was performed according to the manufacturer’s instructions. MiRNA-specific reverse transcription primers and stem-loop quantitative PCR primers are listed in Supplementary Table S2. MiRNA universal SYBR qPCR master mix (MQ101, Vazyme) and ChamQ SYBR qPCR master mix (Q331, Vazyme) were used for quantitative detection of miRNA and lncRNA, respectively. LncRNA quantitative PCR primers are listed in Supplementary Table S2. The formula used to calculate PCR results is the ΔΔCt method (Livak and Schmittgen, 2001), where ΔΔCt = SampleΔCt (Ct Target gene - Ct Reference gene)—ControlΔCt (Ct Target gene - Ct Reference gene). The reference gene used for miRNAs quantification was U6, while the reference gene for lncRNAs was β-actin. The fold change of the target gene is calculated as 2^−ΔΔCt^. For miRNA transfection, synthetic miRNA mimics (Genepharma, Shanghai, China) were introduced into A549T cells using the lipofectamine RNAiMAX transfection reagent (13778075, Thermo Fisher Scientific, Waltham, UK). Cells were cultured after transfection and treated with 20 ng/mL PTX or solvent (negative control) for cell viability or apoptosis testing.

### 2.12 Statistical analysis

The experimental data were analyzed statistically using SPSS (version 22.0, SPSS, Inc., IL, USA) and GraphPad Prism 9 (Graphpad Software, Inc., CA, USA). The results between different groups were expressed as the mean and standard error of mean (SEM) of at least 3 replicates. The initial phase entailed assessing the normal distribution within the data. Upon confirming normal distribution and homogeneity of variance, the *t*-test was applied for the analysis of variance between two groups. Additionally, One-way analysis of variance (ANOVA) and the Bonferroni *post hoc* test were employed for the statistical examination of three or more groups. Unpaired measurement data, deviating from normal distribution or homogeneity of variance, were subjected to analysis through the Kolmogorov-Smirnov test. *p* < 0.05 was used as the threshold of statistical significance. We confirmed that every possible comparison between the study groups was considered.

## 3 Results

### 3.1 Small extracellular vesicles mediate paclitaxel resistance in NSCLC cells

**FIGURE 1:**
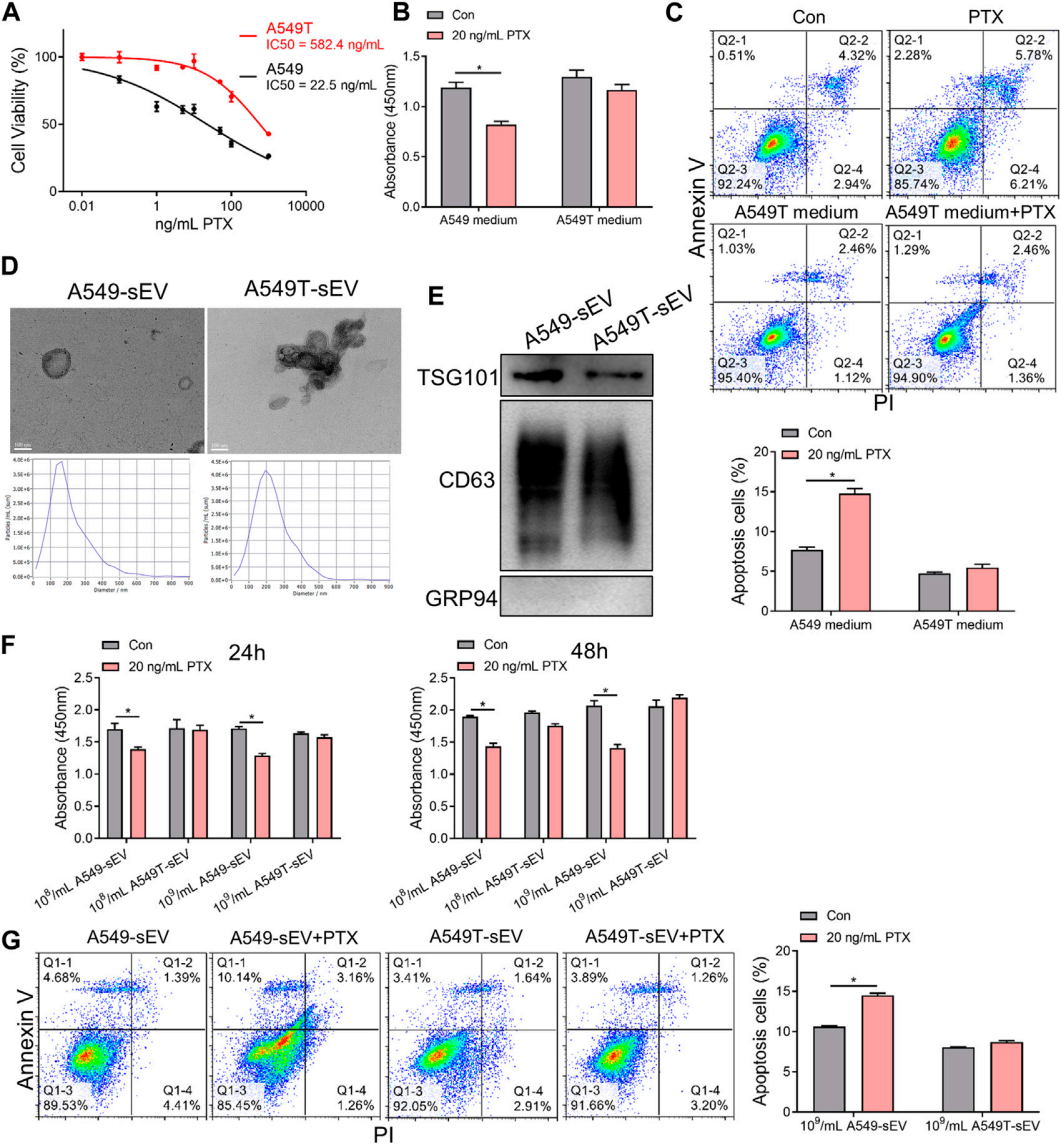
Paclitaxel-resistant A549T cells secrete sEVs to induce resistance in sensitive cells. **(A)** A549 and A549T cells were co-cultured with 0, 0.01, 0.1, 1, 5, 10, 50, 100, and 1000 ng/mL PTX for 48 h. The CCK-8 assay was used to detect cell viability at each concentration, from which half inhibitory concentration (IC50) was calculated. **(B)** A549 cells were treated with A549T-derived medium, or equal volume A549 medium, and 20 ng/mL PTX, or solvent control, for 48 h. Cell viability was measured using the CCK-8 assay. **(C)** The apoptotic rate of A549 cells was measured after 48 h of medium and PTX treatment. **(D)** TEM and NTA were used to evaluate the morphology and particle size distribution of A549 and A549T cell-derived sEVs. Scale bar, 100 nm. **(E)** Western blot was used to detect proteins expression of TSG101, CD63 and GRP94 in sEVs. **(F)** Cells were co-cultured for 24 or 48 h with 20 ng/mL PTX and 10^8^ (or 10^9^) particles/mL of A549 or A549T-derived sEVs. The CCK-8 assay was used to detect cell viability. **(G)** Cells were co-cultured for 48 h with 20 ng/mL PTX and 10^9^ particles/mL of A549 or A549T-derived sEVs, followed by measurement of cell apoptosis rate. All data were normally distributed and variance homogeneity, data are presented as Mean ± SEM, n = 3 (for Western blot and cell apotosis assays) or 5 (for CCK-8 assays); *p < 0.05.

To investigate chemotherapy resistance in NSCLC cells, we selected a small population of Taxol-resistant cells (A549T cells) from NSCLC cell line A549 through continuous PTX co-culture. The results showed that A549T cells were significantly more resistant (IC50 = 582.4 ng/mL) to PTX than A549 cells (IC50 = 22.5 ng/mL, Figure 1A). To determine the effect of paclitaxel-resistant cells on normal NSCLC cells, A549 cells were treated with the conditioned medium from A549T cells treated with PTX. A549 cells preconditioned with A549T medium were resistant to 20 ng/mL PTX, while untreated cells showed lower viability and increased apoptosis after PTX treatment at the same concentration (Figures 1B, C). We hypothesized that A549T might secrete small extracellular vesicles (sEVs) into the conditioned medium, thereby transmitting chemotherapy resistance to A549 cells. We isolated sEVs from A549 and A549T’s medium and characterized the vesicles' morphology and size by transmission electron microscopy (TEM) and nanoparticle tracking analysis (NTA, Figure 1D). Positive expression of CD63 and TSG101 marker proteins, and negative GRP94 expression was identified in these sEVs following the recommended procedures outlined in MISEV2018 guidelines (Alzahrani et al., 2023) (Figure 1E). The sensitivity of the A549 cells to 20 ng/mL PTX treatment was measured after co-culturing with different concentrations of A549T-derived sEVs (10^8^ and 10^9^ particles per mL). We observed these sEVs were internalized by A549 cells (Supplementary Figure S1A), and A549T-derived sEVs significantly enhanced the resistance of A549 cells to PTX after co-culture for 24 and 48 h (Figure 1F). A549 cells alone were sensitive to 20 ng/mL PTX treatment, whereby PTX significantly induced apoptosis. However, 20 ng/mL PTX had little effect on A549 cell apoptosis on cells co-cultured with A549T-derived sEVs (Figure 1G). Western blot results showed that PTX enhanced the activation of caspase 3 in A549 cells with A549-sEV co-culture, but did not increase its activation in cells treated with A549T-sEV (Supplementary Figure S1B). These results suggest that resistant A549T cells can transfer PTX resistance to sensitive cells via sEVs.

### 3.2 Alpha-hederin block small extracellular vesicles-mediated paclitaxel resistance transmission

**FIGURE 2:**
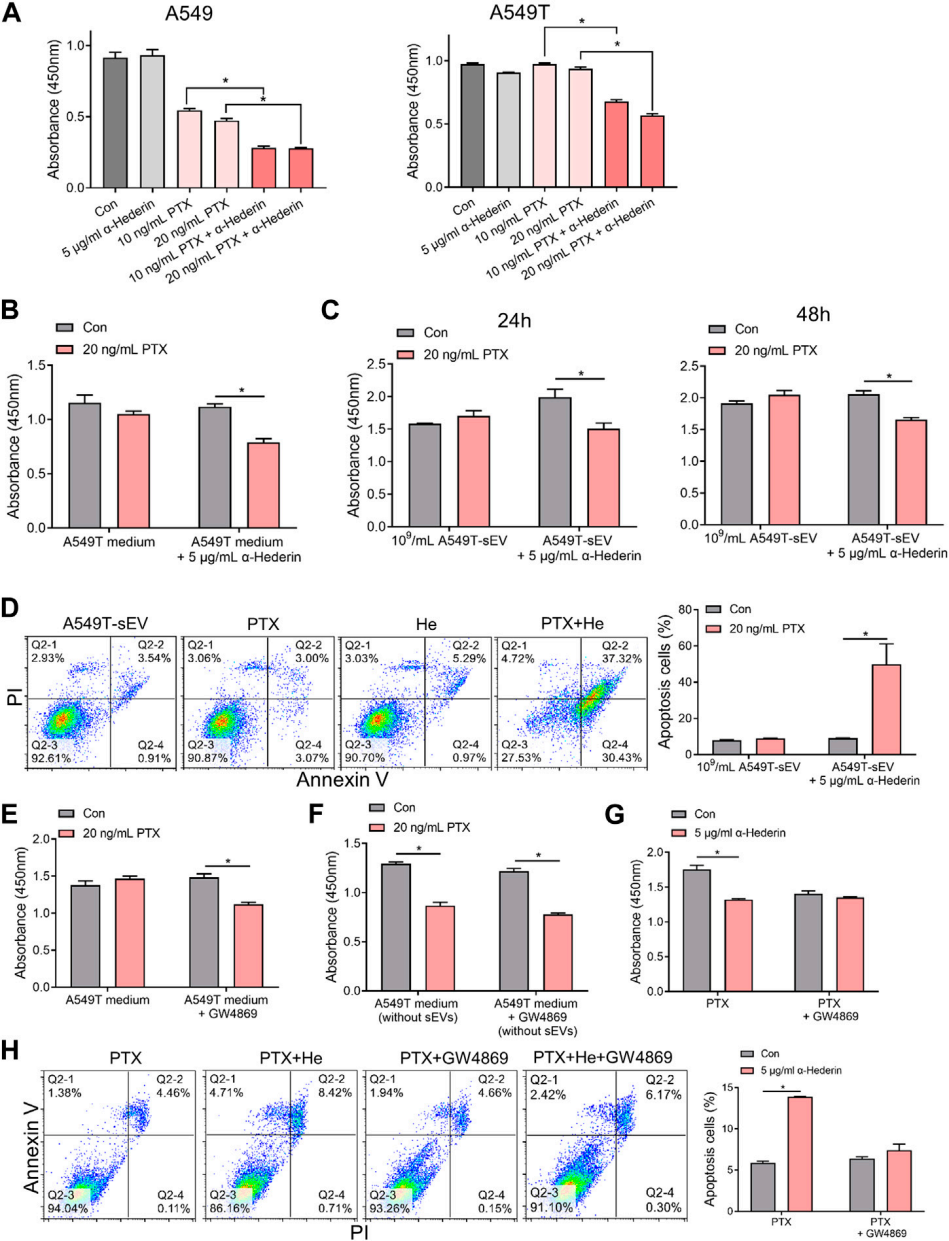
Alpha-hederin blocks small extracellular vesicle-mediated paclitaxel resistance transmission. **(A)** A549 and A549T cells were treated with 5 μg/mL α-hederin, 10 ng/mL PTX, 20 ng/mL PTX, or a combination of both for 48 h. Cell growth and viability were measured using the CCK-8 assay. **(B)** A549 cells were cultured with A549T-derived medium and treated with 5 μg/mL α-hederin, 20 ng/mL PTX, or a combination of both for 48 h. Cell growth and viability were measured using the CCK-8 assay. **(C,D)** A549 cells were cultured with 10^9^ particles/mL of A549T-derived sEVs and treated with 5 μg/mL α-hederin, 20 ng/mL PTX, or their combination for 24 or 48 h. Cell growth and viability were measured using the CCK-8 assay, and cell apoptosis rate was detected. **(E)** A549T cells were treated with 10 μM GW4869 or solvent control for 48 h before obtaining the medium. A549 cells were treated with PTX in this medium for 48 h, and cell viability was measured. **(F)** The A549 cells were treated with PTX in the culture medium from which sEVs had been removed by ultracentrifugation, and cell viability was measured. **(G,H)** A549T cells were treated with 5 μg/mL α-hederin, 20 ng/mL PTX, 10 μM GW4869, or their combinations for 48 h. Cell growth and viability were measured using the CCK-8 assay, and cell apoptosis rate was detected. All data were normally distributed and variance homogeneity, data are presented as Mean ± SEM, n = 3 (for cell apotosis assays) or 5 (for CCK-8 assays); *p < 0.05.

A549 and A549T cells were treated with α-hederin in combination with PTX to explore the effect of α-hederin on NSCLC cells’I sensitivity to PTX therapy. The analysis utilizing the Chou-Talalay method indicated a synergistic sensitizing effect between the two drugs (Supplementary Figure S1C). While concentrations of 5 μg/mL α-hederin did not independently exhibit a significant tumor suppression effect, according to our previous study (Chang et al., 2023), combining α-hederin and PTX was more effective than PTX alone in killing the NSCLC cells (Figure 2A, Supplementary Figures S1D, E). Although 20 ng/mL PTX did not significantly inhibit the proliferation of A549 cells cultured in A549T-derived medium, α-hederin treatment restored the sensitivity of A549 cells to PTX in the same medium (Figure 2B). Interestingly, α-hederin inhibited proliferation and increased apoptosis of A549 cells co-cultured with A549T-derived sEVs upon PTX treatment (Figures 2C, D). To determine whether the α-hederin-mediated sensitivity to PTX treatment depends on sEVs transduction, we used a vesicular secretion inhibitor, GW4869, to block sEVs secretion by A549T cells. A549 cells re-exhibited sensitivity to PTX treatment after co-culture with A549T-derived medium pretreated with GW4869 (Figure 2E), and the removal of sEVs from A549T medium did not affect the cytotoxicity of PTX on A549 cells (Figure 2F). Treatment with α-hederin enhanced the growth inhibition and apoptosis of A549T cells induced by PTX. However, α-hederin had little effect on the responsiveness of A549T cells to PTX in the GW4869-pretreated medium (Figures 2G, H). At the same time, we eliminated the potential impact of α-hederin on the secretion of sEVs in A549T cells, encompassing considerations such as particle number, size and zeta potential (Supplementary Figures S1F–H). Thus, α-hederin blocks PTX-induced resistance transmission through sEVs-related cargo mechanisms.

### 3.3 Paclitaxel-resistant cell-derived sEVs enhance TGFβ and unsaturated fatty acid synthesis pathways

**FIGURE 3:**
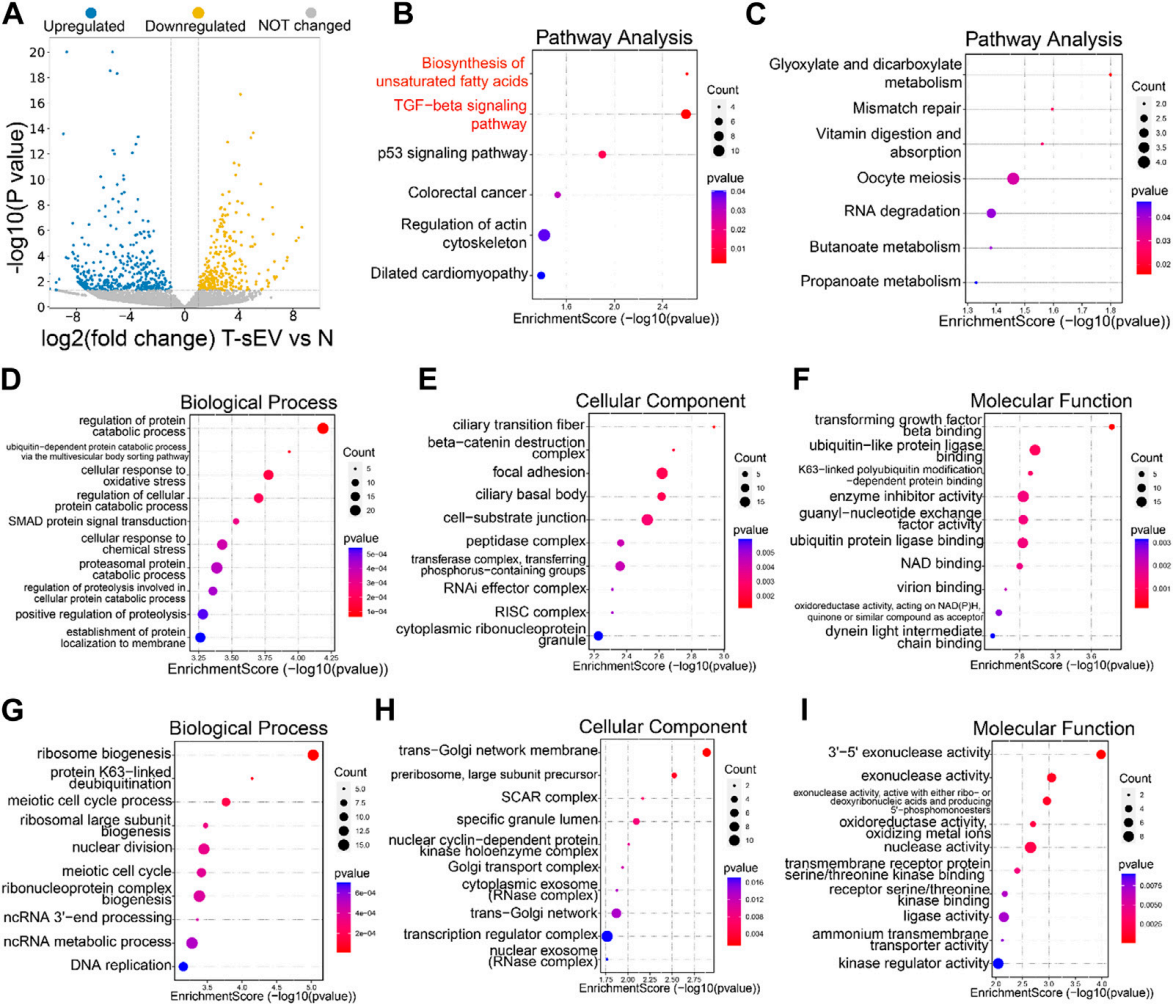
Transcriptomic analysis of A549 cells co-cultured with sEVs derived from resistant cells. **(A)** Volcano plots showing the *p*-value and fold change of differentially expressed mRNAs in the two groups of samples. Upregulated (blue), downregulated (yellow), or unchanged (gray) transcripts in A549T-derived sEVs-treated cells compared with control untreated A549 cells (group N). KEGG signaling pathways enriched with the upregulated **(B)** and downregulated **(C)** genes by sEVs derived from A549T. The dot plot was drawn with *p* < 0.05 as the significance threshold. Gene Ontologies enriched with upregulated **(D–F)** and downregulated **(G–I)** genes, and the dot plot was drawn with *p* < 0.05 as the significance threshold.

We next explored the mechanisms underlying resistance induction by sEVs derived from PTX-resistant A549T cells. RNA sequencing was performed on total RNA extracted from A549 cells co-cultured or not with A549T-derived sEVs. A total of 463 genes were upregulated, and 274 genes were downregulated in A549 cells treated with A549T-derived sEVs compared to untreated cells (Fold change > ± 2.0, *p* < 0.05, Figure 3A, Supplementary Table S1). We used the clusterProfiler package to investigate the biological pathways associated with these differentially expressed transcripts. KEGG analysis showed that sEVs from PTX-resistant cells increased the expression of genes involved in TGFβ signaling and unsaturated fatty acid synthesis and decreased those involved in glyoxylate and dicarboxylate metabolism (Figures 3B, C). Besides, the clusterProfiler package was used to analyze the differentially expressed mRNAs’ Gene Ontology (GO) for biological processes, cell components, and molecular function. PTX-resistant sEVs increased the protein catabolic metabolism process, TGFβ binding signal, and SMAD signal conduction (Figures 3D–F), whereas they reduced ribosome biosynthesis, Golgi membrane transport, and other processes (Figures 3G–I). Based on these results, we assumed that these A549T cells might induce resistance in sEVs-recipient cells mainly by enhancing TGFβ/SMAD signaling and unsaturated fatty acid synthesis pathways.

### 3.4 Alpha-hederin inhibits sEVs-mediated TGFβ/SMAD2 signaling and unsaturated fatty acid synthesis

**FIGURE 4:**
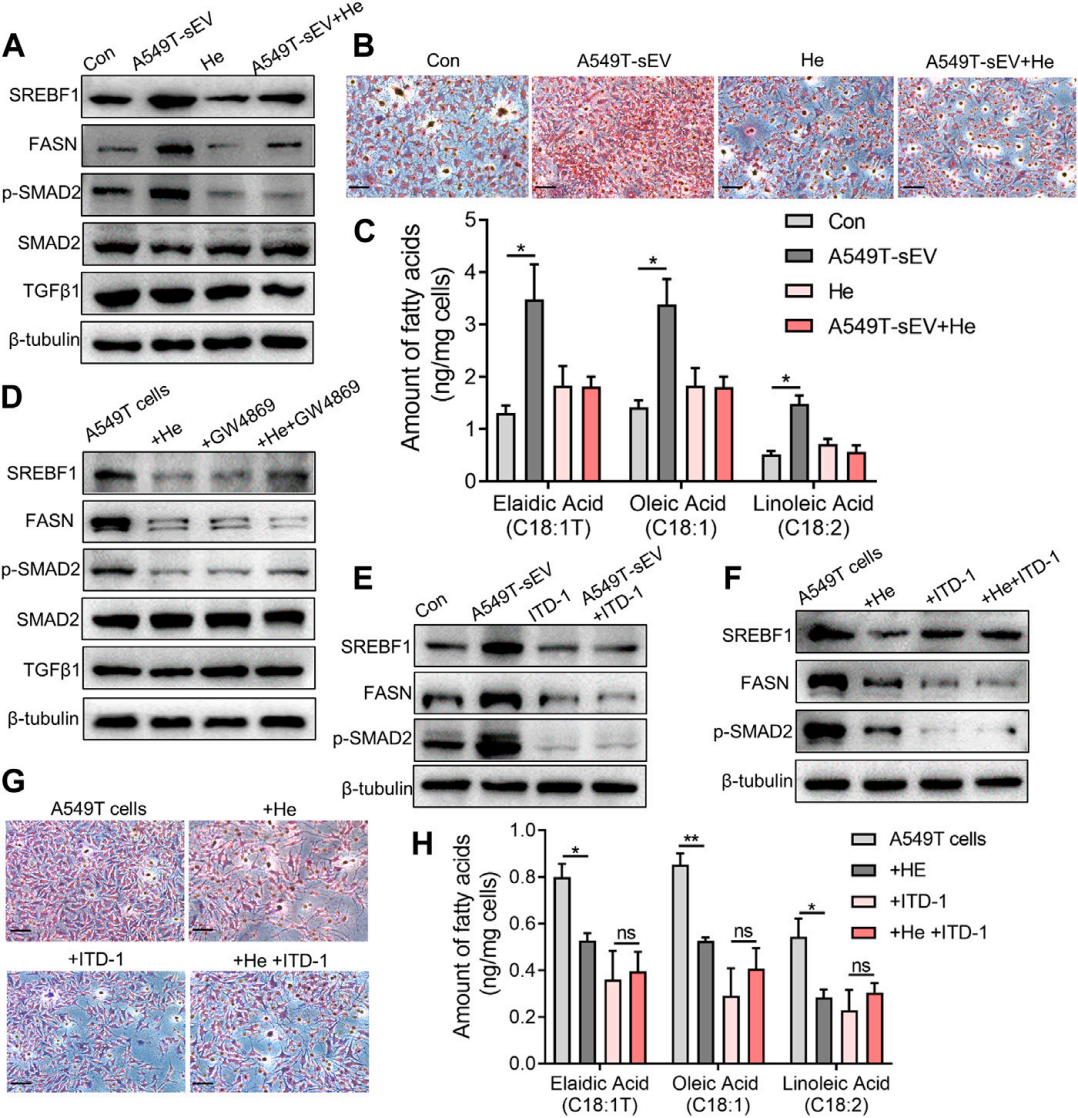
Alpha-hederin inhibits sEVs-mediated TGFβ/SMAD2 signaling and unsaturated fatty acid synthesis. A549 cells were treated with 10^9^ particles/mL A549T-derived sEVs, 5 μg/mL α-hederin, or a combination of both for 48 h. **(A)** Western blot was used to detect proteins related to lipid synthesis and TGFβ signaling. **(B)** Cells were stained with oil red O and photographed. Magnification, ×40; scale bar, 20 μm. **(C)** Cells were collected for targeted fatty acid measurements. The contents of 3 unsaturated fatty acids are shown. **(D)** A549T cells were treated with 5 μg/mL α-hederin, 10 μM GW4869, or their combination for 48 h. Western blot was used to detect proteins related to lipid synthesis and TGFβ signaling. **(E)** A549 cells were treated with 10^9^ particles/mL A549T-derived sEVs, 2 μM ITD-1, or their combination for 48 h. Western blot was used to measure protein expression. **(F)** A549T cells were treated with 5 μg/mL α-hederin, 2 μM ITD-1, or their combination for 48 h. Western blot was used to detect protein expression. **(G)** Cells from **(F)** were stained with oil red O and photographed. Magnification, ×40; scale bar, 20 μm. **(H)** Cells from **(F)** were collected for targeted fatty acid metabolome measurement. The contents of 3 unsaturated fatty acids are shown. All data were normally distributed and variance homogeneity, data are presented as Mean ± SEM, n = 3; *p < 0.05.

A549T-derived sEVs, not A549-derived sEVs, significantly activated TGFβ/SMAD2 signaling and fatty acid synthesis pathway (Figure 4A, Supplementary Figure S2), manifested by increased protein levels of phosphorylated SMAD2, lipid metabolism-related transcription factor SREBF1, and fatty acid synthetase (FASN). However, α-hederin inhibited sEVs-induced SMAD2 phosphorylation and SREBF1 and FASN protein expression. Oil-red O staining data demonstrated that α-hederin impeded lipid accumulation induced by PTX-resistant sEVs in A549 cells (Figure 4B). Targeted metabolomics on 20 unsaturated and 12 saturated fatty acids showed that co-culture with PTX-resistant sEVs increased the accumulation of multiple unsaturated fatty acids (15 out of 20) in A549 cells, including elaidic acid, oleic acid, and linoleic acid. The resistant sEVs promoted the accumulation of some short-chain and medium-chain saturated fatty acids (4 out of 12). The effect of resistant sEVs on fatty acid accumulation disappeared in the presence of α-hederin (Figure 4C, Supplementary Figure S3). Treatment of PTX-resistant A549T cells with α-hederin inhibited SREBF1/FASN expression and SMAD2 phosphorylation. Interestingly, α-hederin had little effect on these proteins in cells whose vesicle secretion was inhibited by GW4869, suggesting that the effect of α-hederin on SREBF1/FASN and TGFβ/SMAD2 signaling was dependent on vesicle secretion (Figure 4D, Supplementary Figure S4A). ITD-1, a selective TGFβ receptor (TGFβR) inhibitor, was used to interrupt TGFβ/SMAD2 signaling in A549T cells and sEVs-treated A549 cells. The results showed that the expression of SREBF1/FASN did not significantly change by A549T-derived sEVs (Figure 4E, Supplementary Figure S4B) or α-hederin (Figure 4F, Supplementary Figure S4C) in ITD-1 treated cells. Treatment with α-hederin enhanced the growth inhibition and apoptosis of A549T cells induced by PTX. However, in A549T cells treated with ITD-1, α-hederin had a slight impact on the therapeutic efficacy of PTX (Supplementary Figures S4D, E). Oil red O staining and targeted fatty acid metabolomics showed that α-hederin reduced lipid and unsaturated fatty acid accumulation in A549T cells, while ITD-1 eliminated these effects (Figures 4G, H, Supplementary Figure S5). Therefore, A549T-derived sEVs upregulated the expression of SREBF1/FASN via TGFβ/SMAD2 signaling, promoting the synthesis and accumulation of unsaturated fatty acids.

### 3.5 Alpha-hederin overcomes sEVs-mediated paclitaxel resistance *in vivo*

**FIGURE 5:**
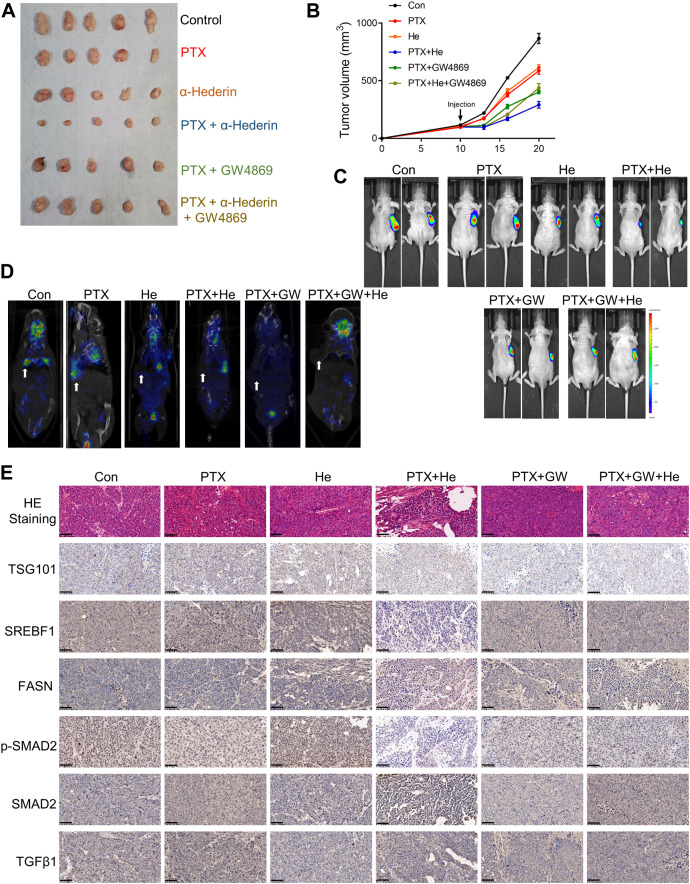
Alpha-hederin sensitizes cells to PTX treatment by modulating sEVs-mediated SREBF1/FASN and TGFβ/SMAD2 pathways activation. **(A,B)** A549T-derived mouse xenograft tumors were treated with control, 3 μg/kg PTX, 5 mg/kg α-hederin, 3 μg/kg PTX +5 mg/kg α-hederin, 3 μg/kg PTX +1 mg/kg GW4869, or 3 μg/kg PTX +5 mg/kg α-hederin +1 mg/kg GW4869 for 9 days. Tumor size and volume were photographed and recorded. **(C)** Representative bioluminescent imaging of luciferase labeled A549T tumor-bearing mice. **(D)** Representative image of micro-FDG PET/CT in tumor-bearing mice. The arrow indicates the location of the tumor. **(E)** HE staining and IHC assays of tumor tissues; scale bar, 50 μm.

We evaluated the effect of alpha-hederin on PTX resistance and its relation to sEVs secretion in NSCLC cells *in vivo*. Firstly, the subcutaneous administration of A549-sEVs in A549 xenograft mice revealed that PTX significantly decelerated tumor growth. However, tumors treated with A549T-sEVs exhibited resistance to PTX therapy (Supplementary Figures S6A, B). Then, while mouse subcutaneous xenograft tumors from A549T cells stably expressing luciferase were resistant to PTX therapy, co-treatment with α-hederin sensitized them to PTX. However, the sensitization effect of α-hederin was blocked by GW4869 (Figures 5A–C). The micro-PET/CT imaging of mice showed that α-hederin and GW4869 significantly inhibited the uptake of the glucose analog ^18^F-FDG in PTX-resistant tumors, suggesting a metabolic regulatory effect of α-hederin and sEVs (Figure 5D). Further, immunohistochemical (IHC) analysis of tumor tissues confirmed that the combination of α-hederin and PTX significantly decreased the expression of SREBF1, FASN, phosphorylated SMAD2, and the sEVs-marker protein TSG101 compared to PTX alone. GW4869 administration showed no significant difference in these proteins between combination therapy (α-hederin and PTX) and PTX alone (Figure 5E, Supplementary Figure S6C). Thus, the therapeutic benefit of α-hederin combined with PTX administration in PTX-resistant tumors is achieved by reprogramming sEVs-related pathways.

### 3.6 Alpha-hederin enhances the activity of miRNA-21-5p, miRNA-23a-3p and miRNA-125b-5p in sEVs and sensitizes cells to paclitaxel therapy

**FIGURE 6:**
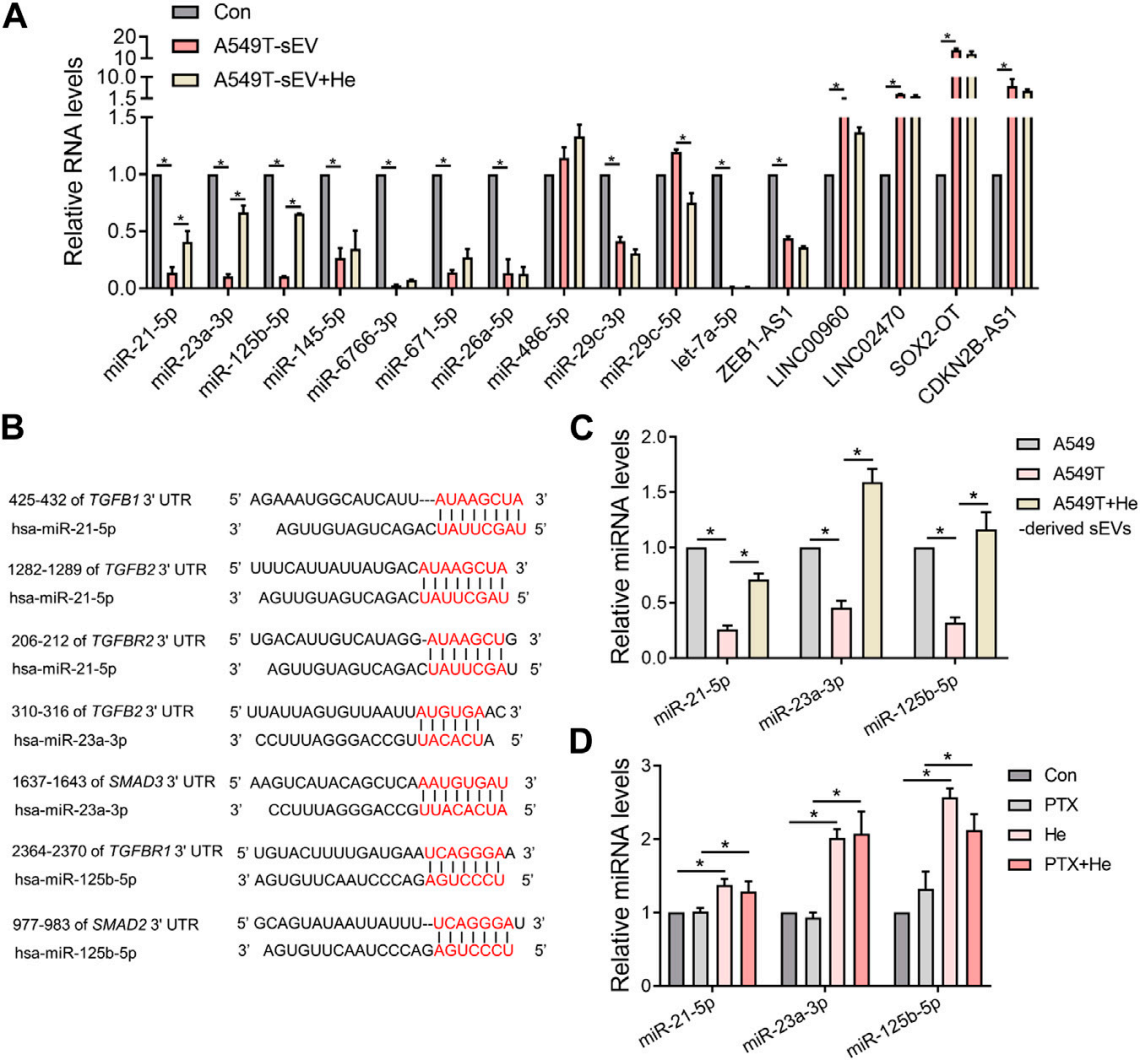
Alpha-hederin therapy enhanced the expression of multiple TGFβ/SMADs-targeting miRNAs in sEVs. **(A)** A549 cells were treated with 10^9^ particles/mL A549T-derived sEVs alone or in combination with 5 μg/mL α-hederin for 48 h. The expression of miRNA and lncRNA targeting TGFβ/SMADs was measured by RT-PCR. **(B)** Targets of miR-21-5p, miR-23a-3p, and miR-125b-5p predicted by the TargetScan database. **(C)** RT-qPCR was used to detect the expression of miR-21-5p, miR-23a-3p, and miR-125b-5p in sEVs derived from untreated A549 cells, untreated A549T cells, and A549T cells treated with 5 μg/mL α-hederin for 48 h **(D)** A549T cells were treated with 5 μg/mL α-hederin, 20 ng/mL PTX or a combination of both drugs for 48 h. RT-qPCR was used to measure miRNA expression. All data were normally distributed and variance homogeneity, data are presented as Mean ± SEM, n = 3; *p < 0.05.

**FIGURE 7:**
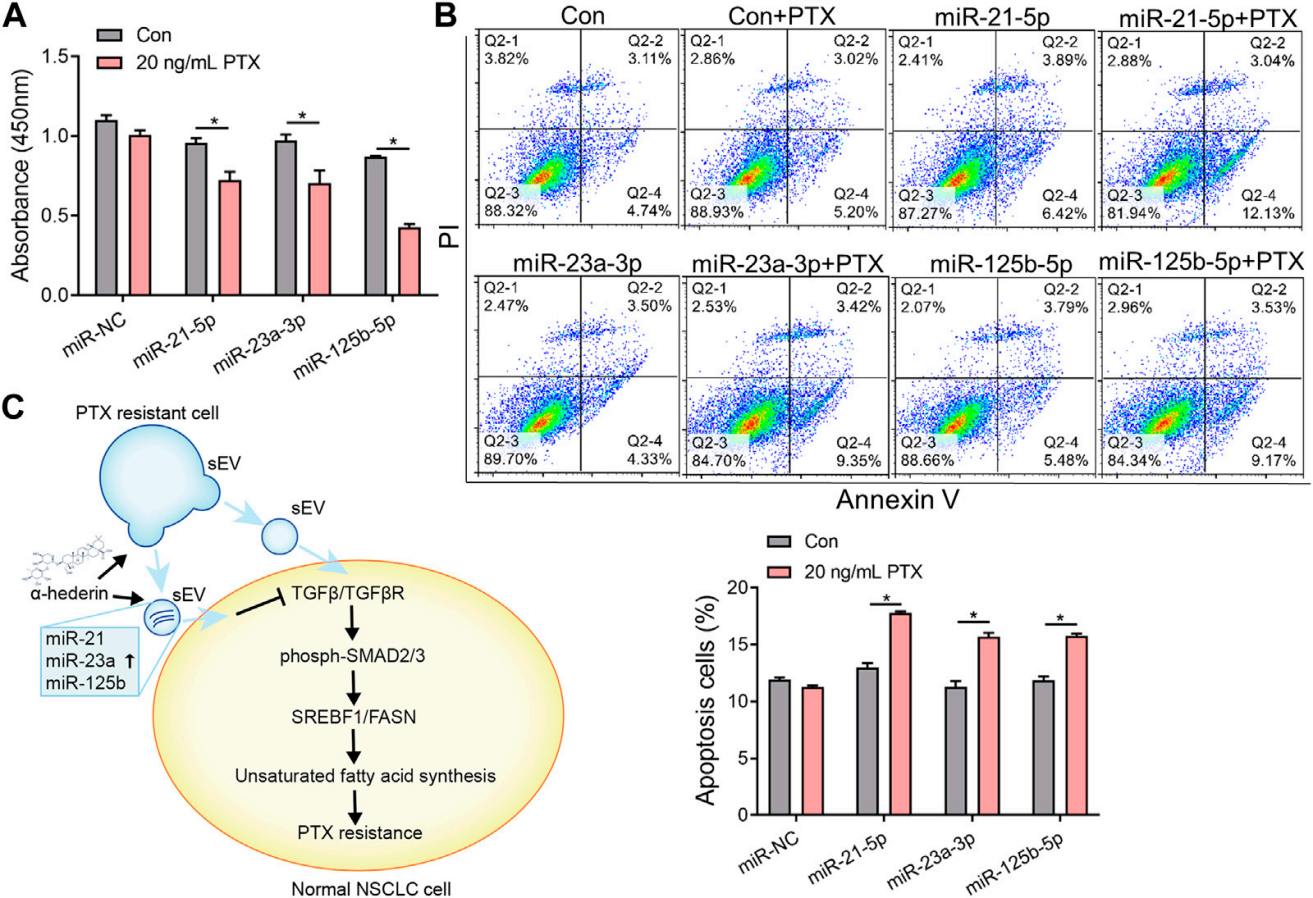
Overexpression of miR-21-5p, miR-23a-3p, or miR-125b-5p increased the sensitivity of resistant cells to PTX therapy. **(A)** A549T cells were transfected with mimics of miR-NC, miR-21-5p, miR-23a-3p, or miR-125b-5p and treated with 20 ng/mL PTX for 48 h. The CCK-8 assay was used to assess cell viability. **(B)** The apoptotic rate of A549T cells treated as in **(A)** was detected. **(C)** Schematic diagram of the mechanism by which α-hederin increased the sensitivity of resistant cells to PTX therapy by reprogramming the miRNA components of sEVs. All data were normally distributed and variance homogeneity, data are presented as Mean ± SEM, n = 3; *p < 0.05.

We explored the mechanisms by which α-hederin overcomes sEVs-mediated PTX resistance. We hypothesized that α-hederin might regulate A549T-derived sEVs cargos involved in TGFβ/SMADs and SREBF1/FASN signaling. A variety of non-coding RNAs, including microRNAs (miR-21-5p, miR-23a-3p, miR-125b-5p, miR-145-5p, miR-26a-5p, miR-6766-3p, miR-671-5p, let-7a-5p, miR-486-5p, miR-29c-3p, and miR-29c-5p) and long non-coding RNAs (lncRNA ZEB1-AS1, LINC00960, LINC02470, lncRNA SOX2-OT, and lncRNA CDKN2B-AS1), were shown to be sorted into sEVs or exosomes and directly targeted regulatory genes in the TGFβ/SMADs pathway (Solé et al., 2015; Fang et al., 2016; Hu et al., 2019a; Huang et al., 2020; Wang et al., 2021b; Chen et al., 2021; Wang et al., 2021c; Lin et al., 2021; Wu et al., 2021; Zhou et al., 2021; Wu et al., 2022a; Luo et al., 2022). Among these non-coding RNAs, we observed that miR-21-5p, miR-23a-3p, and miR-125b-5p decreased after treatment with A549T-derived sEVs and increased after further treatment with α-hederin (Figure 6A). However, the treatment with α-hederin and A549-sEV individually did not have a significant impact on the expression or levels of most miRNAs and lncRNAs (Supplementary Figure S6D). In the TargetScan database, miR-21-5p, miR-23a-3p, and miR-125b-5p were predicted to target the 3′untranslated regions (UTR) of TGFβ (TGFB1, TGFB2), TGFβ receptor (TGFBR1, TGFBR2) and some of the SMAD (SMAD2, SMAD3) family genes (Figure 6B). Western blots and subsequent quantitative analysis results demonstrated that the three microRNAs (miRNAs) exerted an inhibitory effect on the TGFβ1/SMAD2 signaling pathway (Supplementary Figure S6E). The expression of these three miRNAs was significantly lower in sEVs derived from resistant A549T cells than those derived from sensitive A549 cells. In addition, α-hederin treatment significantly increased the level of these miRNAs in sEVs secreted by A549T cells (Figure 6C). At the cellular level, α-hederin alone or combined with PTX increased the expression of the three miRNAs in A549T cells (Figure 6D). Cell viability and apoptosis assays showed that the overexpression of miR-21-5p, miR-23a-3p, or miR-125b-5p enhanced the therapeutic effect of PTX on A549T. Compared with PTX treatment alone, the combination of PTX with either of the miRNAs significantly inhibited the growth of A549T cells (Figure 7A) and increased the proportion of apoptotic cells (Figure 7B). As shown in the schematic diagram in Figure 7C, these results indicate that α-hederin enhanced PTX sensitivity in recipient cells by promoting miR-21-5p, miR-23a-3p, and miR-125b-5p expression and sEVs sorting, and inhibiting TGFβ/SMAD2 and SREBF1/FASN pathways in PTX-resistant cells.

## 4 Discussion

Chemotherapy resistance and recurrence is an important obstacle to NSCLC therapy, and some drugs or targets have been found to help overcome therapeutic resistance. HDAC2 inhibitor ITF2357 reduced the resistance of mutant KRAS NSCLC cells to pemetrexed through a miR-130a dependent mechanism (Cui et al., 2023). Carbonic anhydrase IX inhibitor enhanced the susceptibility of gefitinib and cisplatin-resistant lung cancer cells to iron death (Zhang et al., 2023a). Benzoxy-induced ROS enhanced ohitinib sensitivity by inhibiting the pSTAT3/SOCS3 and KEAP1/NRF2 pathways and promoting mitochondrial dysfunction (Meng et al., 2023). Several multifunctional nanomaterials were used in combination with doxorubicin to overcome multidrug resistance by inducing selective accumulation and mitochondrial damage in tumor cells (Park et al., 2023). In this and previous study, we explained that medicinal plant-derived small molecules shikonin and α-hederin overcome the resistance to cisplatin or paclitaxel in NSCLC cells (Dai et al., 2022).

Tumor resistance is related to sEVs or exosomes, and these vesicles are able to cross biological barriers and provide functional non-coding RNAs (ncRNAs) or proteins to recipient cells. NcRNAs play an important regulatory role in tumor occurrence, development and chemotherapy resistance, and they mainly include miRNAs, lncRNAs, circular RNA, small nucleolus RNAs and transport RNAs (Liu et al., 2019; Wang et al., 2021d). Reduction of sEVs and targeted sEVs therapy restored sensitivity to chemotherapy drugs (Milman et al., 2019). sEVs contents induces the development of drug resistance in some tumor cells, thereby hindering the production and secretion of sEVs, targeting sEVs therapy, and using sEVs as drug delivery vectors are potentially effective means of inhibiting drug resistance.

NSCLC-derived sEVs ncRNAs enhance or inhibit chemotherapy sensitivity. It was found that NSCLC-derived cancer-associated fibroblast cells enhanced cisplatin resistance by transferring sEVs miRNA-130a and miR-20a to recipient NSCLC cells (Zhang et al., 2021; Shi et al., 2022). M2 macrophage-derived sEVs induced cisplatin resistance in lung cancer by regulating the axis of miR-3679-5p/NEDD4L/c-Myc (Wang et al., 2020). For lncRNAs, NSCLC cell-derived sEVs FOXD3-AS1 and SNHG7 activated the PI3K/Akt pathway to promote cancer progression and promote resistance to 5-FU or docetaxel (Mao et al., 2021; Zhang et al., 2022a). sEVs containing upregulated lncRNA H19 promoted erlotinib resistance in sensitive cells via the miR-615-3p/ATG7 axis (Pan and Zhou, 2020). In terms of sEVs-derived circRNAs, circVMP1 promoted cisplatin resistance by modulating the miR-524-5p/METTL3/SOX2 signal axis (Xie et al., 2022). However, several studies have also reported that sEVs-derived ncRNAs may reverse chemotherapy resistance in lung cancer. For example, sEVs miR-193a derived from bone marrow mesenchymal stem cells inhibited the progression of cisplatin-resistant NSCLC by targeting leucine-rich repeat containing protein 1 (Wu et al., 2020a). Circ0000079 blocked the formation of FXR1/PRCKI complex and inhibited the invasion and resistance of NSCLC cells (Chen et al., 2020). Our research outcomes have advanced our comprehension of sEVs-dependent miRNA cargo levels, thus contributing to the understanding of chemotherapy resistance. More importantly, we have introduced pharmacological strategies at the miRNA level to counteract resistance, specifically through the unique modulation of sEVs cargo by α-hederin. The investigation into the mechanism of sEV-mediated PTX resistance is constrained, especially in terms of data related to lung cancer. Previous studies predominantly delved into the mechanistic roles of sEV cargo, including ANXA6, miR-378a-3p, miR-378d, circBACH1, primarily within the context of breast cancer (Yang et al., 2021b; Guo et al., 2021; Xia et al., 2023). Our study has unveiled the transfer of sEVs in the microenvironment of NSCLC between resistant and sensitive cells and has elucidated the mechanism of PTX resistance, thereby substantially enhancing theoretical comprehension in this domain.

Our study, in conjunction with prior research, provides support for the notion that the overexpression of miR-21-5p, miR-23a-3p, and miR-125b-5p contributes to effective tumor treatment through diverse mechanisms, notably by augmenting sensitivity to PTX treatment. The overexpression of miR-21-5p downregulated TGFB1 expression in colorectal cancer cells, triggering cell pyroptosis (Jiang et al., 2020). However, predominant research supports miR-21-5p′s role as an oncogene, as it targeted and inhibited the tumor suppressor gene PTEN, resulting in the degradation of its mRNA (Chen et al., 2018; Zhang et al., 2022b). Our data unveiled that miR-21-5p potentially targeted TGFB1/2, enhancing the sensitivity of NSCLC cells to PTX treatment. This highlights the multifaceted role of miR-21-5p in tumor progression, contingent upon its various target points. MiR-23a targeted and downregulated the SMAD3 signal, inhibiting tumor epithelial-mesenchymal transition and progression (Liu et al., 2016; Wang et al., 2017). With TGFβ/SMAD2/3 as the focus, our data suggested that miR-23a-3p had a sensitizing effect on PTX treatment. However, when miR-23a targeted SNHG5, it exhibited an antagonistic role, increasing resistance of ovarian cancer to PTX treatment (Lin et al., 2020). The upregulation of miR-125b served as a pivotal factor in attenuating the malignant characteristics of tumors through the downregulation of SMAD2 expression (Zhou et al., 2015). Importantly, our investigations and corroborating evidence from earlier study highlight the significant anti-tumor effects of miR-125b-5p when transported via sEVs or exosomes to recipient tumor cells (Kim et al., 2021). Meanwhile, miR-125b-5p targeting the tumor suppressor STARD13 exhibited a pro-metastatic role in the exosomes secreted by pancreatic cancer (Wu et al., 2020b). This implies that these miRNAs assume varied roles in tumor progression contingent upon distinct targets. The engagement of more intricate functionalities in enhancing the sensitivity of PTX treatment necessitates further elucidation.

Several studies have shown that α-hederin regulated the cell proliferation, cycle, apoptosis and autophagy in various cancer cells and drug-resistant cells (Cheng et al., 2014; Sun et al., 2019). In NSCLC cells, our previous study demonstrated that α-hederin promoted iron death by disrupting the glutathione redox system (Wu et al., 2022b). α-Hederin have been found to significantly regulate tumor metabolism, by inhibiting glycolysis and the expression of GLUT1, HK2, PKM2 and LDHA (Fang et al., 2021). α-Hederin induced excessive reactive oxygen species in mitochondria, sensitising several tumor cells to death signals (Zhan et al., 2018). SREBF1 and FASN, as important checkpoints of lipid metabolism, have been suggested to mediate tumor therapy resistance (Ma et al., 2021; Li et al., 2023). α-Hederin has been shown to exhibit pronounced anti-tumor effects or enhance responses to chemotherapy, providing promise for clinical translation. For example, α-hederin, sourced from natural plants, is easily accessible and extractable (Belmehdi et al., 2023). It demonstrates excellent biocompatibility and cell permeability, and its combination with nano-drugs can enhance the effectiveness of tumor delivery (Nicol et al., 2017). The consumption of medicinal plants containing α-hederin through dietary intake contributes to overall health improvement and disease prevention, including tumors (Butt and Sultan, 2010). Nevertheless, before α-hederin can be successfully translated into clinical applications, several challenges must be addressed. Presently, α-hederin is predominantly studied at the cellular level, lacking precise evidence regarding safe dosages in animal research. Further investigation is needed to explore its pharmacokinetic parameters.

This study possesses certain limitations. Firstly, the small sample size in our experiment (n = 3 or 5) may limit the sensitivity of the study results. This underscores the need for selecting larger and more representative sample sizes in subsequent research to enhance credibility. Our exploration of sEVs’ effects was limited to a pair of resistant-sensitive cells (A549T-A549), with a lack of broader cell line studies, particularly those involving different genotypes or phenotypes. Expanding our investigation to incorporate a more diverse array of resistant cell lines, especially those resistant to targeted therapies or immune treatments, would enhance the depth of our findings and potentially offer clinical advantages. Furthermore, while sEVs derived from PTX-resistant cells were identified as drivers of the TGFβ signaling and SREBF1 metabolic pathways, there could be additional sEVs cargo beyond miRNAs involved in this process. A more in-depth analysis of RNA, proteins, or metabolites within sEVs is essential for a comprehensive understanding of their contents. Investigating how α-hederin influences the sorting of these cargoes into sEVs is also a topic that warrants further exploration.

## 5 Conclusion

In summary, this study demonstrated that α-hederin could overcome sEVs-dependent resistance to PTX therapy in NSCLC cells *in vivo* and *in vitro* by enriching sEVs in miRNAs targeting TGFβ/SMADs signaling. Our results help provide a new strategy for treating NSCLC, particularly α-hederin, which potentially can reverse chemotherapy resistance in NSCLC.

## Data Availability

The datasets presented in this study can be found in online repositories. The names of the repository/repositories and accession number(s) can be found in the article/Supplementary Material.
