# Supplementary material for: Alpha-hederin reprograms multi-miRNAs activity and overcome small extracellular vesicles-mediated paclitaxel resistance in NSCLC
Source: Front Pharmacol. 2024 Feb 1;15:1257941. doi: 10.3389/fphar.2024.1257941 (PMC10867254; doi:10.3389/fphar.2024.1257941)
Supplement: Supplementary file 3 [file DataSheet1.DOCX]

**Supplemental Figure S1**

**
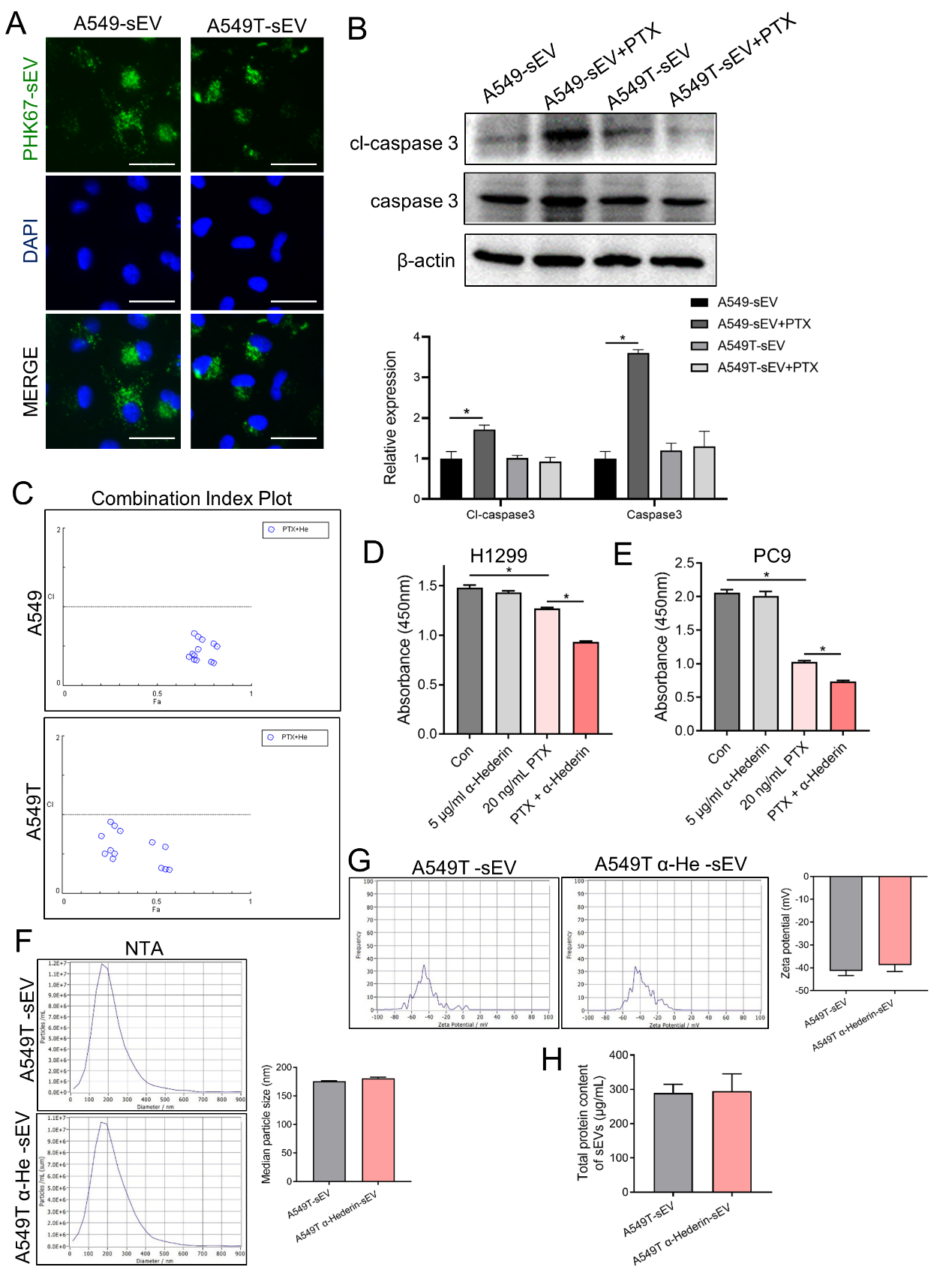
**

**(A)** Fluorescence microscopy of A549 cells after being incubated PKH67-labeled (green) sEVs. Cell nuclei were stained with DAPI (blue). Scale bar, 100 μm.

**(B)** Cells were co-cultured for 48 hours with 20 ng/mL PTX and 10^9^ particles/mL of A549 or A549T-derived sEVs, followed by western blot detection for caspase 3 and cleaved (cl)-caspase 3 expression. Quantitative analysis data of the density for the western blot bands was depicted, normalized to β-actin.

**(C)** CompuSyn software based on the classic Chou-Talalay method was used to evaluate the combined effects of PTX and α-hederin in A549 and A549T cells. The Combination Index Plot graph illustrated that a combination index below 1 indicated a synergistic sensitizing effect between the two drugs.

**(D E)** H1299 and PC9 cells were treated with 5 μg/mL α-hederin, 10 ng/mL PTX, 20 ng/mL PTX, or a combination of both for 48 h. Cell growth and viability were measured using the CCK-8 assay.

**(F)** 2 × 10^8^ A549T cells were treated with 5 μg/mL alpha-hederin and control solvent for 72 hours, respectively, and collected 50 mL of culture medium. The sEVs from these culture medium were extracted using an ultracentrifugation method, resulting in a final volume of 1 mL in PBS. NTA were used to evaluate particle size distribution of A549T cell-derived sEVs.

**(G)** The ZETAview instrument was employed to assess the zeta potential of sEVs.

**(H)** Protein quantification assays were used to evaluate the total protein content of sEVs. * P < 0.05.

**Supplemental Figure S2**

**
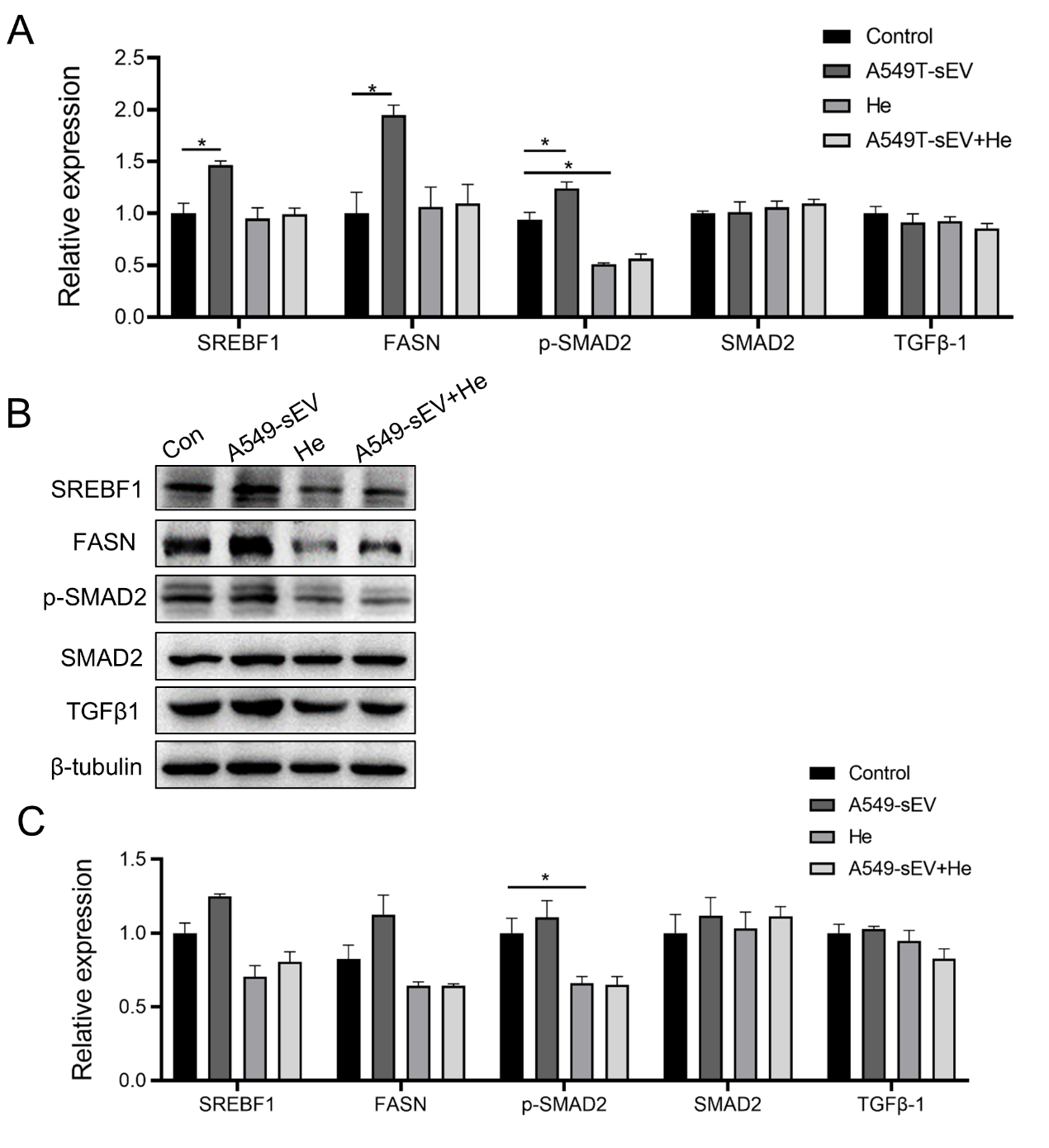
**

**(A)** Quantitative analysis data of the density for the western blot bands depicted in Figure 4A, normalized to β-tubulin.

**(B)** A549 cells were treated with 10^9^ particles/mL A549-derived sEVs, 5 μg/mL α-hederin, or a combination of both for 48 hours. Western blot was used to detect proteins related to lipid synthesis and TGFβ signaling.

**(C)** Quantitative analysis data of the density for the western blot bands depicted in Figure S2B, normalized to β-tubulin. * P < 0.05.

**Supplemental Figure S3**


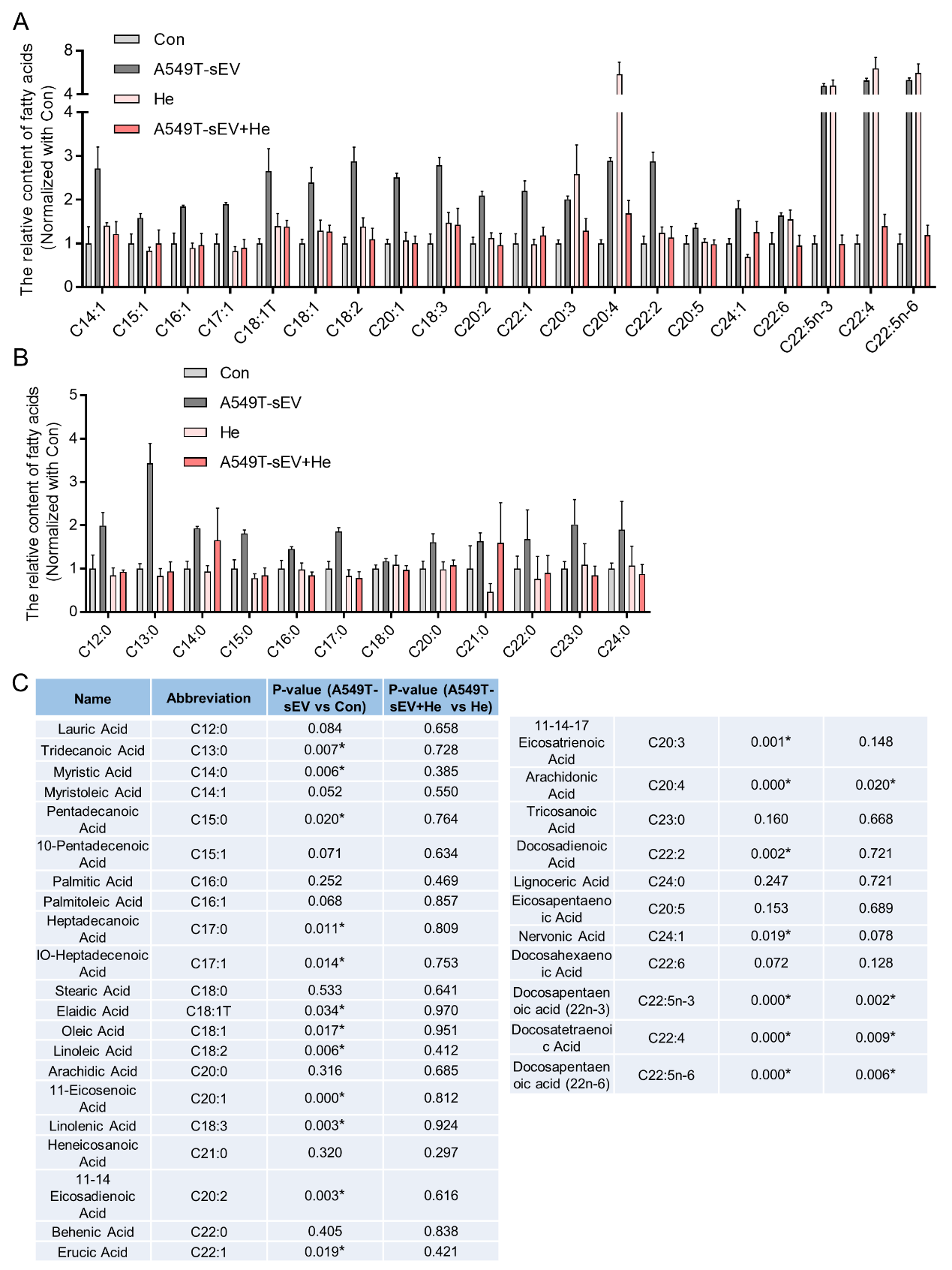


A549 cells were treated with 10^9^ particles/mL A549T-derived sEVs, 5 μg/mL α-hederin, or a combination for 48 h. Targeted fatty acid metabolome was performed to detected the contents of unsaturated **(A)** and saturated **(B)** fatty acids. The statistical differences and P-values of each fatty acid content between groups were shown in panel C. * P < 0.05.

**Supplemental Figure S4**

**
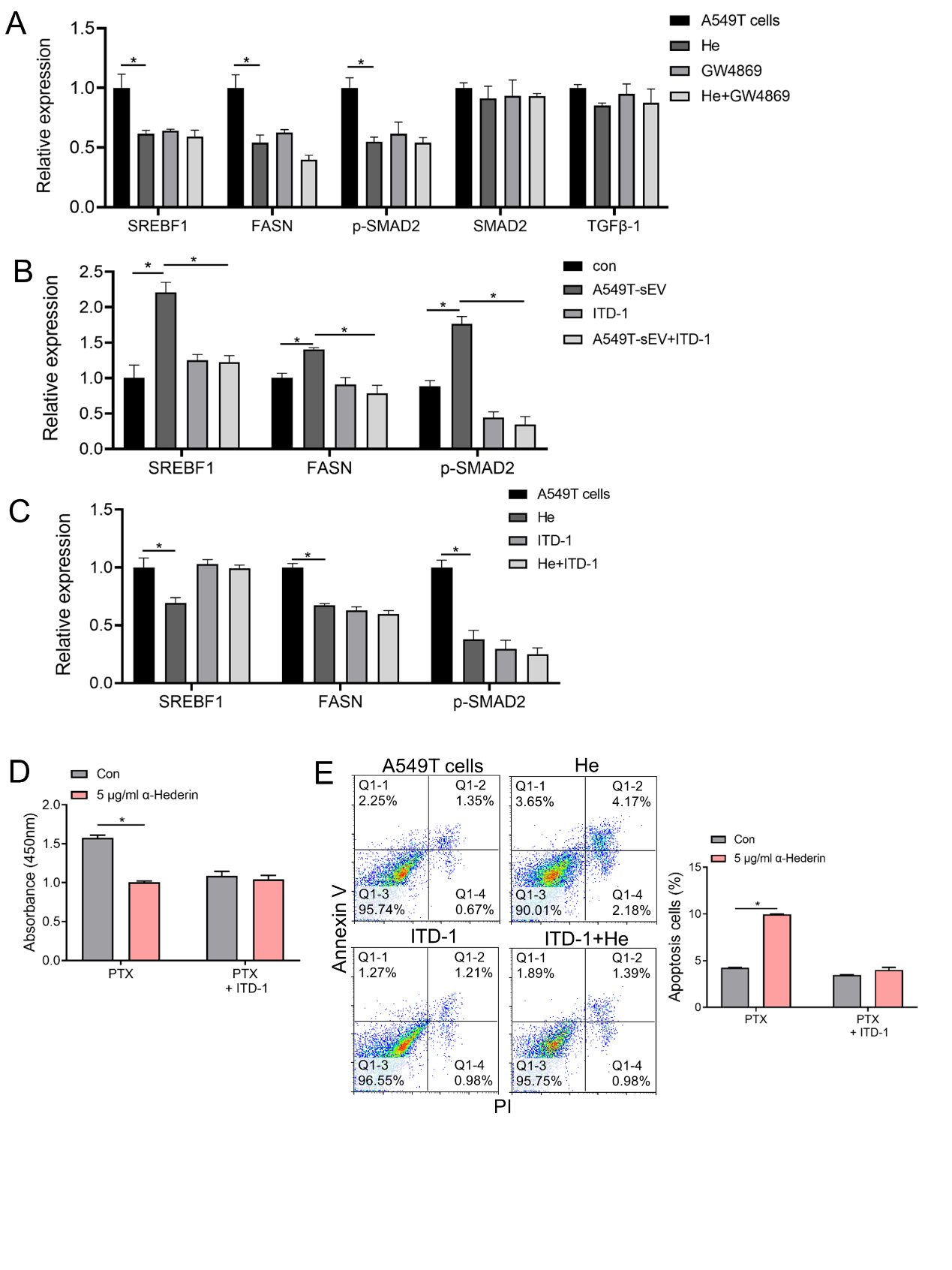
**

**(A-C)** Quantitative analysis data of the density for the western blot bands depicted in Figure 4D/E/F, normalized to β-tubulin.

**(D, E)**A549T cells were treated with 5 μg/mL α-hederin, 20 ng/mL PTX, 2 μM ITD-1, or their combinations for 48 hours. Cell growth and viability were measured using the CCK-8 assay **(D)**, and cell apoptosis rate was detected **(E)**. * P < 0.05.

**Supplemental Figure S5**


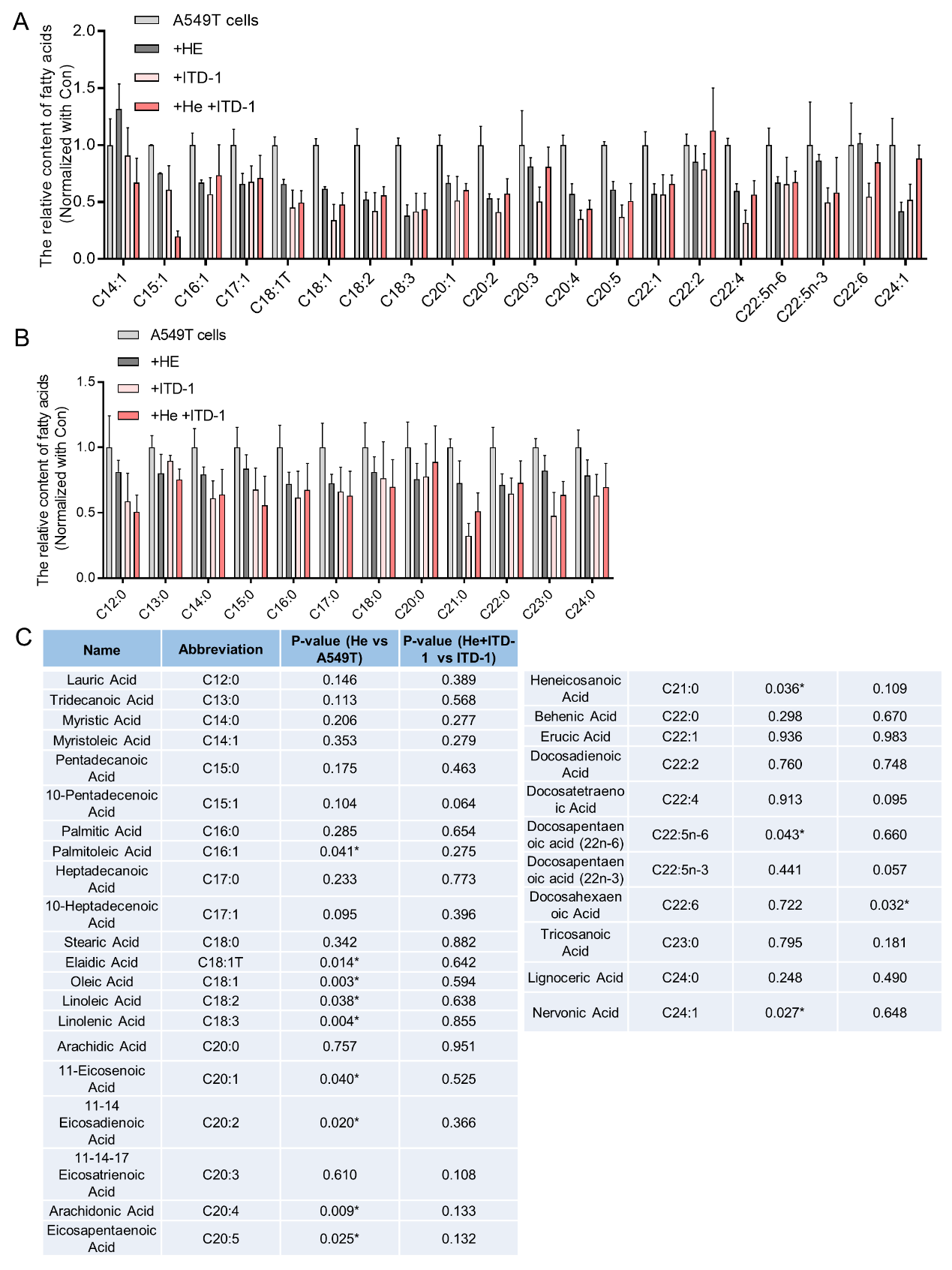


A549 cells were treated with 10^9^ particles/mL A549T-derived sEVs, 2 μM ITD-1 or a combination for 48 h. Targeted fatty acid metabolome was performed to detected the contents of unsaturated **(A)** and saturated **(B)** fatty acids. The statistical differences and P-values of each fatty acid content between groups were shown in panel C. * P < 0.05.

**Supplemental Figure S6**


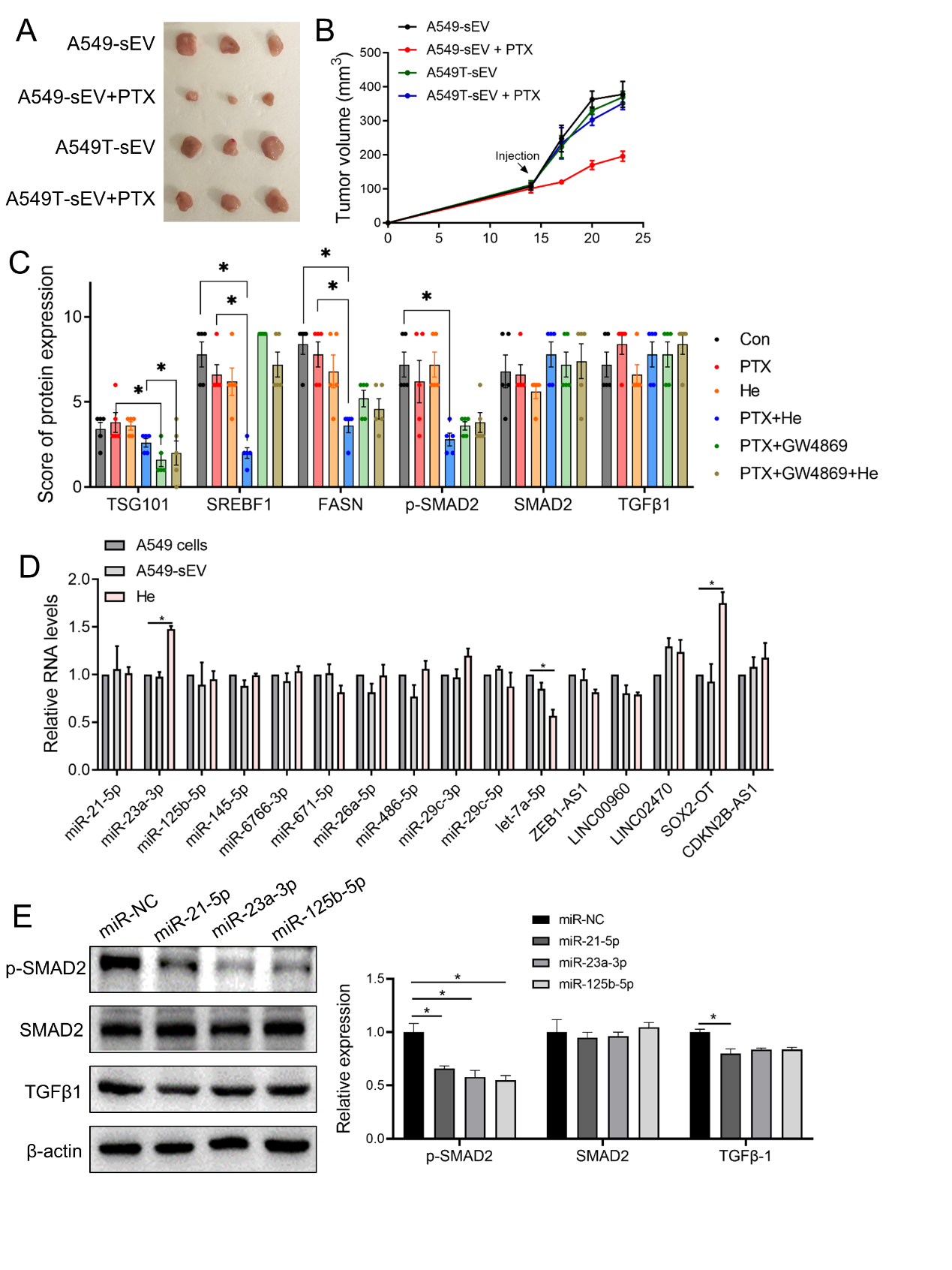


**(A B)** Subcutaneous inoculation of 5 × 10^6^ A549 cells was performed in nude mice. Once the tumor volume reached 100 mm^3^, we administered A549-sEVs or A549T-sEVs through subcutaneous injections at a dose of 10 μg per administration every 3 days. Simultaneously, intraperitoneal injections of PTX were administered at a dose of 3 μg/kg every 3 days and 0.1% DMSO as control. Tumor size and volume were photographed and recorded.

**(C)** Quantitative analysis of the IHC staining in Figure 5E was performed. Kolmogorov-Smirnov test was performed, n=5.

**(D)** A549 cells were treated with 10^9^ particles/mL A549-derived sEVs and 5 μg/mL α-hederin for 48 hours. The expression of miRNA and lncRNA targeting TGFβ/SMADs was measured by RT-PCR.

**(E)** Mimics of miR-21-5p, miR-23a-3p, miR-125b-5p, and the control miR-NC were transfected into A549T cells for 48 h. Western blot was used to detect proteins related to TGFβ signaling. Quantitative analysis data of the density for the western blot bands was depicted, normalized to β-actin. * P < 0.05.

**Supplemental Materials and Methods**

**PKH67-lablled sEVs**

A549 or A549T-derived sEVs resuspended in PBS were labelled with green fluorescent membrane dye PKH67 (PKH67GL, Sigma-Aldrich, MO, USA). Cells were treated with 40 μg/mL sEVs labelled PKH67 in six-well plates at 37°C for 24 hours. Then, slides were washed three times using PBS, fixed with 4% formaldehyde for 20 minutes, followed by three additional PBS washes. Cell nuclei were stained by DAPI fluorescent stain (D9542, Sigma-Aldrich). Images were taken using a fluorescence microscope (Olympus, London, England).

**MiRNA and Target Prediction**

The TargetScan database employs 3P-seq tag technology to discern the binding of miRNA to the 3'UTR region of transcripts. This process involves comparing the obtained data with the pre-existing 3'UTR annotations in the NCBI database, thereby furnishing the 3'UTR sequences associated with miRNA. In particular, we entered the names of specific miRNAs to retrieve targeted transcripts. It has been noted that certain gene transcripts within the TGFβ/SMADs pathway bind with these miRNAs, with a higher absolute value of the Context++ score signifying a stronger binding affinity.

**sEV isolation and characterization according to MISEV 2018**

For sEV isolation, we meticulously verified the absence of contaminants in the source cells, ensuring a cell viability exceeding 98%. The composition of the DMEM medium, including glucose, glutamine, and antibiotic levels, was meticulously assessed to guarantee their non-interference with sEV secretion and content composition. During the experimental phase, sEV extraction was carried out using medium from non-cultured cells to unequivocally confirm the absence of vesicles. In terms of sEV characterization, the post-dilution sample concentration for NTA detection ranged from 10^6 to 10^9 particles/mL. Both NTA size analysis and TEM images revealed that the majority of vesicles exhibited sizes smaller than 200nm. The total protein concentration of sEV, as determined by a standard curve, consistently fell within the linear range. For the western blot characterization of sEV, we specifically opted for the transmembrane protein CD63, the ESCRT complex component TSG101, and the intracellular protein GRP94 for detection.
